# High rate of spontaneous inhibitor clearance during the long term observation study of a single cohort of 524 haemophilia A patients not undergoing immunotolerance

**DOI:** 10.1186/1756-8722-6-63

**Published:** 2013-08-30

**Authors:** Giuseppe Tagariello, Alfonso Iorio, Davide Matino, Donata Belvini, Roberta Salviato, Roberto Sartori, Paolo Radossi

**Affiliations:** 1 Transfusion Service, Hemophilia and Regional Blood Disease Centre, Castelfranco Veneto Hospital, ULSS 8 Regione Veneto, Treviso, Italy; 2 Health Information Research Unit, Department of Clinical Epidemiology and Biostatistic and Hemophilia Clinic, Department of Medicine, McMaster University, Hamilton, ON, Canada; 3 Experimental Medicine and Biochemical Sciences, University of Perugia, Perugia, Italy

**Keywords:** Hemophilia A, Inhibitors, ITI

## Abstract

**Background:**

The natural history of inhibitors in patients with haemophilia A not undergoing immune tolerance induction (ITI) is largely unknown. A recent randomized controlled trial suggests that the higher the FVIII dose used for ITI, the faster the clearance and the lower the rate of bleeding, without any difference in the rate of tolerance. We aimed at assessing the rate of spontaneous inhibitor clearance in a large cohort of patients not undergoing ITI.

**Methods:**

A retrospective analysis of anti-FVIII inhibitors of long-term registry data in a single centre cohort of 524 haemophilia A patients considered for synovectomy was performed. Patients were tested for inhibitors before and 15 days after any and each surgical episode and thereafter did not undergo immune tolerance at any time.

**Results:**

The cumulative incidence of inhibitors overall was 34% (180 out of 524) with the highest percentage of 39% (168 out of 434) in severe patients which represented 83% of the cohort. Among the 180 inhibitor patients: 63 had permanent inhibitors; 70 fulfilled current criteria for transient inhibitors but a third category of 47 additional patients cleared the alloantibody spontaneously in >6 months. At logistic regression, both the inhibitor titre and the gene mutation were shown to predict time to clearance.

**Conclusions:**

Spontaneous clearance of inhibitors over variable time in the absence of ITI treatment was found in up to 2/3 of the cases.

## Introduction

Haemophilia A treatment, consisting of the administration of factor VIII (FVIII) concentrates, can be complicated in a variable percentage of cases (0-50%) by the occurrence of inhibitor antibodies, which render treatment ineffective [1-4]. A proportion of these antibodies thereafter disappear; antibodies cleared within 6 months are known as 'transient' while those lasting longer are called 'persistent' and are deemed to continue throughout the patient’s life [5,6]. With the aim of eradicating the inhibitor, these patients are treated with high dose FVIII, referred to as 'immune tolerance induction' (ITI). ITI positively affects the life of haemophilia patients by reducing the time to inhibitor eradication and the bleeding rate while on treatment [7], but its widespread adoption made and makes it very difficult to understand the natural history of inhibitors.

Very little is actually known about the natural long term history of inhibitors in absence of ITI, even though a seminal observation of spontaneous resolution of inhibitors in patients who continued to be exposed to standard doses of FVIII was reported by Rizza and Matthews [8]. More recent work has indicated the possibility that non-tolerized inhibitors may present different patterns over time including not only 'stable positivity' or 'stable negativity', but also a third category named 'unstable' [9]. A better understanding of the natural history of inhibitors not undergoing ITI is essential to clarify the still unknown immunological mechanism underlying ITI [10] and to appropriately evaluate its efficacy [11,12], which is particularly important in view of the huge costs of this treatment (in the region of £2,000,000 per treatment in 2003) [13]. In fact, the estimate of the proportion of spontaneously resolving inhibitors in the haemophilia population is critical, jointly with evidence that ITI speeds up the process of spontaneous tolerance more than it affects the rate of inhibitor clearance [7], to understand the role for ITI as compared to alternative management strategies.

With the specific purpose of further clarifying the natural history of inhibitors in terms of clearance rate and time, a single centre retrospective study was performed on a prospectively followed cohort of non-tolerized, mostly genotyped patients with haemophilia A and inhibitors.

## Methods

### Study population

A cohort of patients from one centre (Castelfranco Veneto Haemophilia Centre) where surgical synovectomy was adopted, in the 70s and 80s, as the main treatment method and where inhibitor patients had never undergone ITI.

This was a database originated research. Data were extracted anonymously from the database of clinical and laboratory data of the Hemophilia Center in Castelfanco Veneto. A generic consent to store information for research purposes was obtained from the patients over time, but, for the need of gathering a complete inception cohort, no specific consent was sought for this specific retrospective study protocol.

Haemophilia A was diagnosed and classified according to factor VIII plasma levels [14]. Inhibitor titre was assessed by the Bethesda assay based on measuring the amount of FVIII inactivated by patient plasma mixed in equal proportions with pooled normal plasma as a source of FVIII [15]. For the purpose of this analysis inhibitors were considered 'low' if the titre was < 5 BU/ml and 'high' if it was > 5 BU/ml and patients accordingly defined as low and high responders respectively.

The cohort consisted of all consecutive patients admitted to the centre from 1973–2010 who were tested for inhibitors the first time and at least 5 more times during a minimum 24-month follow up period and 3-month minimum interval between each test. Patients were regularly tested for inhibitors before and 2 weeks after surgical synovectomy.

Patients with less than 5 tests were excluded. Exposure days before inclusion in the study cohort ranged from 0 to >200 while all the inhibitor negative patients reached 200 exposure days during follow up. The entire cohort was treated with factor concentrates approved and available at the time, i.e. cryoprecipitate at the beginning, then plasma derived concentrates manufactured from Italian plasma (by FarmaBiagini and subsequently Kedrion, as Koate, Uman-Cry and Emoclot) and lately recombinant concentrates (used in a minority of patients). Bypassing agents were used to treat bleedings in patients with high inhibitor titre. When patients reached a low inhibitor level (= < 5 BU), they were treated with FVIII concentrates for spontaneous bleeding or for surgery. In these cases, FVIII was dosed calculating a neutralizing dose (BU × 40 × kg/bw) and adding it to the dose needed for the targeted increment in the patient plasma factor VIII level (frequently in the range of 30–50 IU kg/bw). When treated, patients were closely monitored for any anamnestic response by laboratory testing every other day for 1 or 2 weeks. Most of the increases in titer occurred within an average of 6 days (range 3–10).

### Time-dependent classification of inhibitors

Inhibitors were classified as follows: 'transient' if the inhibitor disappeared spontaneously within 6 months and did not reappear when the patient was exposed to factor VIII; 'slowly resolving' if the inhibitor disappeared spontaneously after 6 months and did not reappear when exposed to factor VIII; 'persistent' if the inhibitor remained positive or could not be detected in the absence of factor VIII treatment but reappeared when factor VIII was re-administered.

### Mutation analysis

From the Italian Registry of Haemophilia A Mutations [16], it was possible to identify patient mutations, classified accordingly as, inversions (int1inv and int22inv), null mutations (large deletions, nonsense and frameshift ins/del) or not null mutations (all the others).

### Statistical analysis

To estimate and compare the probability of inhibitor resolution, we have used a model of clinical outcome (class of inhibitor) as predicted by immunological response (high versus low) and gene mutation (inversion, null, not null) using a multinomial logistic regression.

## Results

### Patients

**Figure 1:**
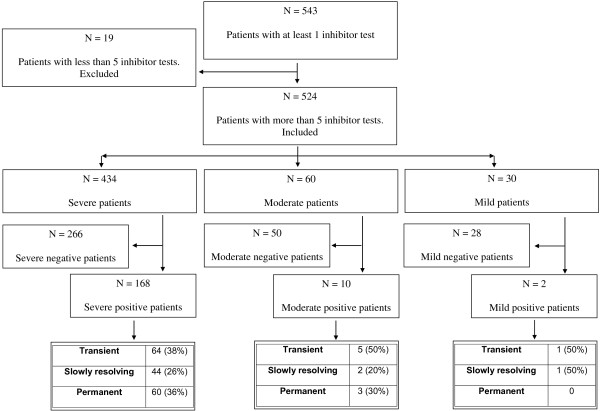
Flow chart of the cohort of patients seen at Castelfranco Veneto Haemophilia Centre, 1973–2010.

The total number of patients was initially 543. They were all tested for inhibitors at least once but 19 patients, with less than 5 tests, were excluded from the analysis (Figure 1).

### Inhibitors

**Figure 2:**
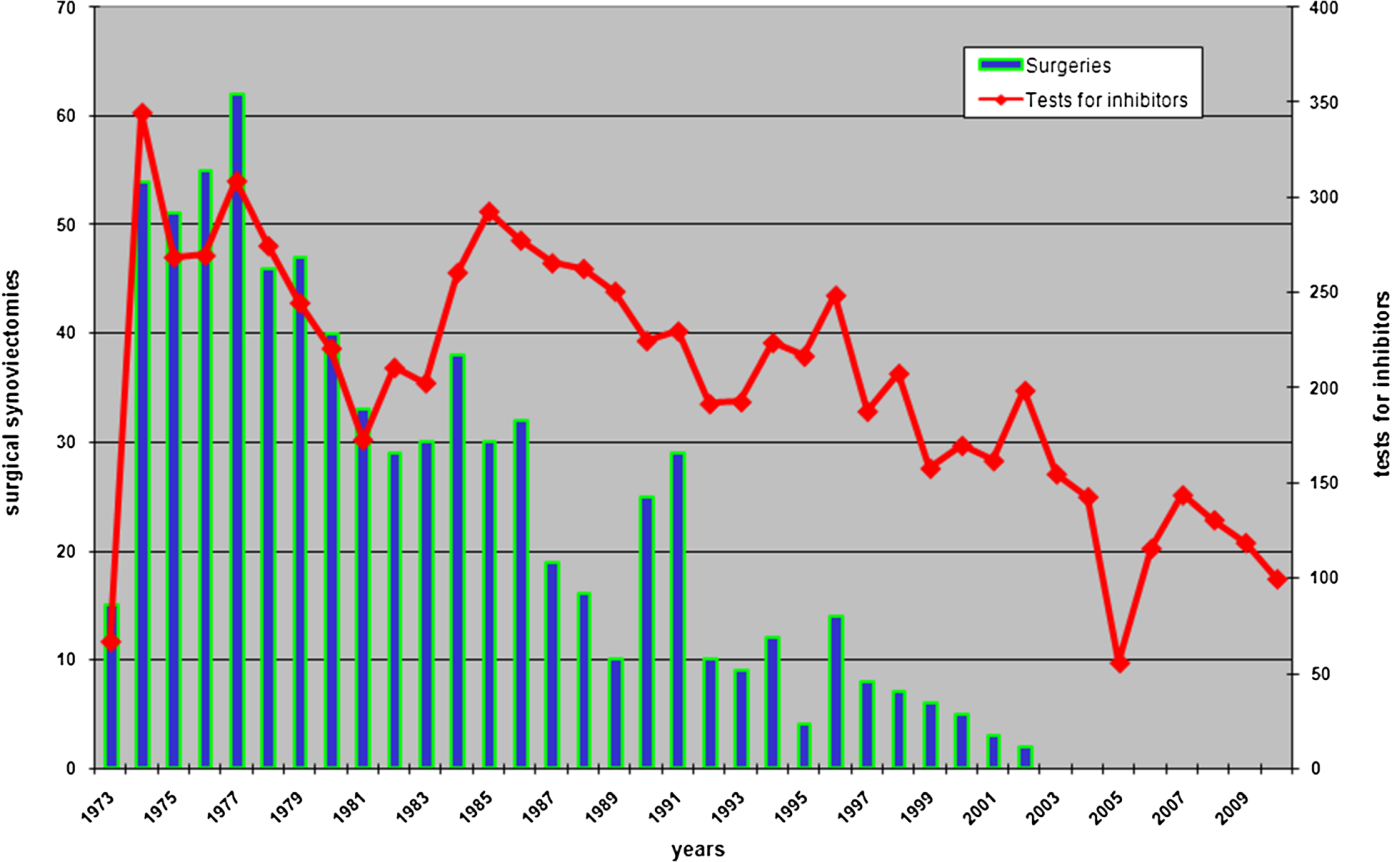
**The figure shows tests for inhibitors (upper curve) and surgical synovectomy performed at Castelfranco Veneto Haemophilia Centre, 1973–2010.** The majority of the tests were performed during the period 1973–1990 when most of the surgical synovectomy programme was developed. The curve shows two peaks, the first during the 70s when the synovectomy programme was more intensive and the second during the mid-80s when patients undergoing synovectomy were overlapped by patients regularly followed up for inhibitor. The last two decades represent the follow up of the patients (right upper curve) and more recent years when surgical synovectomy was completely abandoned and substituted by synoviorthesis (data not reported).

In total, 7,779 test results were available with a median of 15 tests per patient (Figure 2). The mean (+/− SD) age at the onset of inhibitors was 17+/−8 yrs (range 3–47). The mean (+/− SD) follow up was 24+/−12 yrs (range 8–36).

The cumulative inhibitor incidence total was 34% (180/524), 39% (168/434) in severe cases, 16% (10/60) in moderate ones and 7% (2/30) in mild patients. The distribution of inhibitors in persistent, slowly resolving and transient according to haemophilia severity and inhibitor characteristics is reported in Figure 1 and Table 1. 29 inhibitor patients were found positive when first tested, of these, 22 were high responders and 7 low.

**Table 1:** Distribution of patients with and without inhibitor development, high and low responders, transient, slowly resolving and permanent, dependent on the severity of the disease

	**Severe**	**Moderate**	**Mild**	**Total**	**High responders**	**Low responders**	**OR**
	**n = 434**	**n = 60**	**n = 30**	**n = 524**	**n = 79**	**n = 101**	(*)
Without inhibitor	266	50	28	344			
With inhibitor	168	10	2	180			
Transient	64 (38%)	5 (50%)	1 (50%)	70 (39%)	8 (10%)	62 (61%)	-
Slowly resolving	44 (26%)	2 (20%)	1 (50%)	47 (26%)	15 (19%)	32 (32%)	3.6
Permanent	60 (36%)	3 (30%)	0	63 (35%)	56 (71%)	7 (7%)	62

The condition of HR at the onset confers the highest risk of persistent inhibitor (56 out of 79, 71%) while only a minority of the patients become persistent when the onset is as LR (7 out of 101, 7%).

(*)The OR represents the risk of having a permanent or slow resolving inhibitor for those being HR as compared to those being LR.

### Mutations

Mutations were identified in 335/524 patients (64%). Transient, slowly resolving, persistent inhibitor distribution and titre is shown in Table 2. The presence of inversions/null mutations was higher in the persistent and high responding inhibitor groups. The median persistence time of spontaneously clearing slowly resolving inhibitors is reported in Table 3.

**Table 2:** Distribution of patients with and without inhibitor, high and low responders, transient, slowly resolving and permanent dependent on the mutation type

**N° of patients**	**NULL mutations inversions (Int1/22)**	**NULL mutations other**	**NOT null mutations**	**Unknown mutation**	**Total**
	**N = 156**	**N = 79**	**N = 91**	**N = 198**	**N = 524**
Without inhibitor	87	40	68	149	344
With inhibitor	69	39	23	49	180
Transient	17 (25%)	14 (36%)	10 (44%)	29 (59%)	70 (39%)
Slowly resolving	20 (29%)	6 (15%)	7 (30%)	14 (29%)	47 (26%)
Permanent	32 (46%)	19 (49%)	6 (26%)	6 (12%)	63 (35%)
High Responders	38 (55%)	21 (54%)	7 (30%)	13 (27%)	79 (44%)
Low Responders	31 (45%)	18 (46%)	16 (70%)	36 (73%)	101 (56%)

**Table 3:** Inhibitor resolution (years) in patients with slowly resolving type inhibitors, dependent on inhibitor type response

	**High responders**	**Low responders**	**Total**
N° patients	15	32	47
Median (years)	6	3	5
Average (years)	12	7,8	8,7

### Logistic regression analysis

The characteristics of the models and analysis details are reported in Table 4 and its legend. Since underlying gene mutation data was known in only a proportion (131/180) of inhibitor patients the probability of developing a transient, slowly resolving or permanent inhibitor was estimated in two different ways. If available, the probability associated with both the initial inhibitor titre and the gene mutation was assessed, otherwise only the inhibitor titre was used.

**Table 4:** Multinomial univariate logistic regression

**Multinomial model**	**Transient**	**Slowly resolving**	**Permanent**
Inibitor titre §	1	3.63	62.0
SE	-	1.78	34.06
Pseudo R-Square	0.24		
Chi2	94.26		
P	<0.001		
Inibitor titre §	1	1.76	32.83
SE	-	1.03	19.11
Inversions #	1	1.57	1.85
SE	-	0.94	1.40
Nullmutations #	1	0.60	1.43
SE	-	0.41	1.15
Pseudo R-Square	0.24		
Chi2	66.65		
P	<0.001		

Model A – Inhibitor titre (180 cases).

Model B Inhibitor titre and gene mutation (131 cases).

§Regression coefficient for high titre versus low titre.

#Regression coefficient versus not null mutations.

We fitted two separate multinomial logistic regression models: one, in the entire population, assessing the probability of developing a transient, slowly resolving or permanent inhibitor associated with the initial inhibitor titre (model A), and another, in those with the mutation available, assessing the risk associated with both the initial inhibitor titre and the gene mutation (model B), even if the model with the gene mutation alone was not significant (probably due to insufficient sample size). We assumed as baseline risk the patient with low titre inhibitor in model A and the patient with low titre inhibitor and not null mutation in model B.

In model A, the relative risk ratio for a patient with a high responding over one with low responding inhibitor to have a slowly resolving inhibitor is 3.63 (95% CI 1.39 – 9.47) and to have a permanent inhibitor is 62 (95% CI 21.12 – 182.00). The relative risk ratio for a permanent over a slow resolving was 17.1 (95% CI 9.02 – 25.11). The predicted probabilities estimated by the model and used to calculate the relative risk ratio are reported in table.

In model B, after adding the gene mutation, the relative risk ratio for a patient with a high responding over one with low responding inhibitor to have a permanent inhibitor over a transient is 32.8 (95% CI 21.5 – 44.1) and over a slowly resolving is 18.6 (95% CI 10.1 – 27.1), with no significant effect associated to the gene mutation. The predicted probabilities estimated by the model and used to calculate the relative risk ratio are reported in table. The full parametric model with the interactions between inhibitor titre and gene mutation (Log likelihood −102.122, LR chi2(10) = 76.87, Prob > chi2 = 0.0000, Pseudo R2 = 0.2734) showed that the effect of the titre was not statistically significant when considered alone; on the contrary, in patients with inversions compared to those with not null mutations a statistically significant and clinically relevant increase in the risk for permanent inhibitors in patients with high titre inhibitors (21.7, 95% CI 1.80 – 263) was found, together with a statistically significant but not clinically relevant increase in patients with low titre inhibitors (0.03,95% CI 0.001–0.73).

The relative risk ratio between a high and low responder in having a slowly resolving inhibitor is 3.63 (95% CI 1.39 – 9.47) and a permanent inhibitor 62 (95% CI 21.12 – 182.00). In the full model - inhibitor titre, gene mutation and their interactions, the effect of the titre was not statistically significant when considered alone, whereas, in patients with inversions compared to those with non null mutations a statistically significant and clinically relevant increase in the risk for permanent inhibitors in patients with high titre inhibitors (21.7, 95% CI 1.80 – 263) was found. It was not possible to assess the effect of large mutations in this study due to their relatively low incidence.

## Discussion

This work reports the natural history of inhibitors in a cohort of patients followed up for more than 35 years, receiving FVIII replacement therapy but never ITI. In 64% of the patients the inhibitor was spontaneously cleared over a period of up to 12 years. As to risk factors for inhibitors, the study confirms that null mutations are strong predictors of inhibitor development, and it adds that the combination of inhibitor titre and gene mutation type is a possible predictors of spontaneous inhibitor remission. The role of these results in understanding the mechanism and impact of ITI on the natural history of inhibitors requires further study.

### Strengths of the study

The patients were tested every time they underwent surgical synovectomy or exposure to factor VIII for bleeding at the optimal time of two weeks after FVIII administration making the monitoring ideal. The strict inhibitors testing was necessary to avoid the risk of uncontrolled bleeding during synovectomy. The results of this close observation has made feasible a study, based on a cohort subjected to stringent follow up and well-documented inhibitor incidence from as far back as the 70s and 80s, when inhibitor testing was not routinely performed in all Haemophilia Centres.

Due to the early implementation of this strategy, it has been possible to detect all the patients with inhibitors, including low titre as well as transient ones and to identify subgroups of patients on the basis of different time-dependent inhibitor clearance. On the opposite, inhibitor surveillance programs started worldwide as recombinant products became available and were not as suitable to assess the natural history of inhibitors because in the meanwhile ITI has become widespread used.

### Limitations of the study

The value of this retrospective analysis has to be considered mainly exploratory, and some unaccounted confounders might have played a role. Furthermore, as studies on ITI do not have an internal control group of patients not undergoing ITI, any inference from the natural history of inhibitors to ITI treated patients and vice-versa can only be considered as indirect. Some characteristics of the population deserve mention. First, the high rate of surgical synovectomy that occurred in this study might have affected the rate of inhibitor appearance and disappearance. Second, the mean patient age at inhibitor development was rather high, which is explained by the fact that the study in question goes back to the 70s and 80s, a period when concentrates were still not widely available or extensively used. Third, the intensity of treatment spun over a very high range and was not used to adjust the analysis. How these population characteristics could have affected the results and impair their external validity remains to be assessed.

### The natural history of inhibitors: incidence

The cumulative inhibitor incidence in the cohort appears relatively high (34%), particularly so if compared with reports from the same period, but it is in keeping with similar studies conducted later on in other settings [17-19] and substantially overlapping the results from the recent prospective RODIN study [20].

The possibility of a higher incidence of inhibitors in our cohort because of the surgery cannot be excluded as it has been hypothesized that tissue damage would trigger the immune system, raising the risk of developing inhibitors [21], but the same hypothesis has not been confirmed in another case–control study [22].

Another possible explanation for this higher incidence is that the inhibitor rate reported in previous studies was incorrectly low. The low inhibitor rate in the 1980s could actually be explained, as suggested by Iorio et al. [18], by the combined effect of the year of the studies (the older the study, the lower the rate, and no study before the 1980s was included) and the testing frequency. As discussed above, the cohort underwent very frequent inhibitor testing, even when clinical inefficacy was not suspected.

### The natural history of inhibitors: clearance

In addition to 38% of transient inhibitors, 26% of slowly resolving inhibitors disappeared in a median of 3 to 6 years, respectively, in low and high responders. The characteristics of the patients spontaneously clearing their inhibitors are of interest, particularly when contrasted with available evidence from ITI studies [7,23-25]. First, the upper bound of ITI duration needed to obtain tolerance is considered to be up to 33 months, close to the 3-year median time period for spontaneous resolution of the inhibitors among the low responders in our study cohort. Second, a low inhibitor titre has been recognized as significant predictive factor for success of ITI, as it has shown to be in our subgroup of slowly disappearing inhibitors, in which 90% of low titre patients spontaneously lose the inhibitor. Third, underlying haemophilia genotype has been associated with the risk of anti-FVIII inhibitor development [26-28] and the probability of ITI success [29,30]; most patients with large deletions undergoing ITI fail to respond whereas patients with int22inv have an intermediate and variable success rate. The logistic regression analysis performed in our study, though limited by the small number of available patients and incomplete accounting for all potential confounders, seems to fit this picture.

## Conclusions

This long term observational study showed that the prevalence of patients with truly persistent inhibitors is 1/3, while 2/3 of inhibitors may resolve spontaneously These figures largely overlap with results obtained in the ITI study cohorts [7,23-25]. The implications of these results may vary depending on the clinical and societal setting (i.e. the range of treatments available and affordable). If confirmed in controlled clinical observations, more likely to happen in resource poor settings where ITI is not yet standard of care, these results pave the way to a risk stratification strategy for the selection of treatment in patients with inhibitors.

## Competing interests

The authors declare that they have no competing interests.

## Authors’ contributions

GT and AI designed the study, interpreted data and drafted the manuscript; DM performed the statistical analysis critically revised the draft manuscript; DB, RS, RS and PR collected and interpreted data and critically revised the draft manuscript. All authors read and approved the final manuscript.

## References

[B1] Gill FM The natural history of factor VIII inhibitors in patients with hemophilia A Prog Clin Biol Res 1984 150 19 29 6431427

[B2] Hay CR The epidemiology of factor VIII inhibitors Haemophilia 2006 12 Suppl 6 23 8 17123390 10.1111/j.1365-2516.2006.01362.x

[B3] Wight J Paisley S The epidemiology of inhibitors in haemophilia A: a systematic review Haemophilia 2003 9 4 418 35 10.1046/j.1365-2516.2003.00780.x 12828678

[B4] Darby SC Keeling DM Spooner RJ Wan Kan S Giangrande PL Collins PW Hill FG Hay CR UK Haemophilia Centre Doctors' Organisation The incidence of factor VIII and factor IX inhibitors in the hemophilia population of the UK and their effect on subsequent mortality, 1977–99 J Thromb Haemost 2004 2 7 1047 54 10.1046/j.1538-7836.2004.00710.x 15219185

[B5] Lusher JM Inhibitors in young boys with haemophilia Baillieres Best Pract Res Clin Haematol 2000 13 3 457 468 10.1053/beha.2000.0088 11030045

[B6] Rothschild C Gill J Scharrer I Bray G Transient inhibitors in the Recombinate PUP study Thromb Haemost 2000 84 1 145 146 10928491

[B7] Hay CR Dimichele DM The principal results of the International Immune Tolerance Study: a randomized dose comparison Blood 2012 119 6 1335 1344 10.1182/blood-2011-08-369132 22101900

[B8] Rizza CR Mattews JM Effects of factor VIII replacement on the level of FVIII antibodies in haemophiliacs Br J Haematol 1982 52 1 13 24 10.1111/j.1365-2141.1982.tb03857.x 6810911

[B9] Caram C De Souza RG De Sousa JC Araújo Pereira T Do Amaral Cerqueira AM Van Der Bom JG Rezende SM The long-term course of factor VIII inhibitors in patients with congenital haemophilia A without immune tolerance induction Thromb Haemost 2011 105 1 59 65 21057702 10.1160/TH10-04-0231

[B10] Waters B Lillicrap D The molecular mechanisms of immunomodulation and tolerance induction to factor VIII J Thromb Haemost 2009 7 9 1446 1456 10.1111/j.1538-7836.2009.03538.x 19583822

[B11] Berntorp E Astermark J Baghaei F Bergqvist D Holmström M Ljungberg B Norlund A Palmblad J Petrini P Stigendal L Säwe J Treatment of haemophilia A and B and von Willebrand's disease: summary and conclusions of a systematic review as part of a Swedish health-technology assessment Haemophilia 2012 18 2 158 65 10.1111/j.1365-2516.2011.02723.x 22151198

[B12] Srivastava A Brewer AK Mauser-Bunschoten EP Key NS Kitchen S Llinas A Ludlam CA Mahlangu JN Mulder K Poon MC Street A Treatment Guidelines Working Group The World Federation Of Hemophilia. Guidelines for the management of hemophilia Haemophilia 2013 19 1 e1 47 10.1111/j.1365-2516.2012.02909 22776238

[B13] Knight C Paisley J Wight J Jones ML Economic modelling of different treatment strategies for haemophilia A with high-responding inhibitors Haemophilia 2003 9 4 521 540 10.1046/j.1365-2516.2003.00783.x 12828681

[B14] White GC Rosendaal F Aledort LM Lusher JM Rothschild C Ingerslev J on behalf of the Factor VIII and Factor IX Subcommittee Definitions in Hemophilia Thromb & Haemost 2001 85 3 560 11307831

[B15] Kasper CK Aledort LM Counts RB Edson JR Fratantoni J Green D Hampton JW Hilgartner MW Lazerson J Levine PH McMillan CW Pool JG Shapiro SS Shulman NR Van Eys J A more uniform measurement of Factor VIII inhibitors Thromb Diath Haemorrh 1975 34 2 869 872 1198543

[B16] Margaglione M Castaman G Morfini M Rocino A Santagostino E Tagariello G Tagliaferri AR Zanon E Bicocchi MP Castaldo G Peyvandi F Santacroce R Torricelli F Grandone E Mannucci PM AICE-Genetics Study Group The Italian AICE-Genetics hemophilia A database: results and correlation with clinical phenotype Haematologica 2008 93 5 722 728 10.3324/haematol.12427 18387975

[B17] Ehrenforth S Kreuz W Scharrer I Linde R Funk M Güngör T Krackhardt B Kornhuber B Incidence of development of factor VIII and factor IX inhibitors in haemophiliacs Lancet 1992 339 8808 594 8 1347102 10.1016/0140-6736(92)90874-3

[B18] Iorio A Halimeh S Holzhauer S Goldenberg N Marchesini E Marcucci M Young G Bidlingmaier C Brandao LR Ettingshausen CE Gringeri A Kenet G Knöfler R Kreuz W Kurnik K Manner D Santagostino E Mannucci PM Nowak-Göttl U Rate of inhibitor development in previously untreated hemophilia A patients treated with plasma-derived or recombinant factor VIII concentrates: a systematic review J Thromb Haemost 2010 8 6 1256 1265 10.1111/j.1538-7836.2010.03823.x 20345722

[B19] Hay CR Palmer B Chalmers E Liesner R Maclean R Rangarajan S Williams M Collins PW on behalf of United Kingdom Haemophilia Centre Doctors' Organisation (UKHCDO) Incidence of factor VIII inhibitors throughout life in severe hemophilia A in the United Kingdom Blood 2011 117 23 6367 6370 10.1182/blood-2010-09-308668 21471523

[B20] Gouw SC van der Bom JG Ljung R Escuriola C Cid AR Claeyssens-Donadel S Van Geet C Kenet G Mäkipernaa A Molinari AC Muntean W Kobelt R Rivard G Santagostino E Thomas A van den Berg HM PedNet and RODIN Study Group Factor VIII products and inhibitor development in severe hemophilia A N Engl J Med 2013 368 3 231 239 10.1056/NEJMoa1208024 23323899

[B21] Gouw SC Van Der Bom JG Van Den M Berg H Treatment-related risk factors of inhibitor development in previously untreated patients with hemophilia A: the CANAL cohort study Blood 2007 109 11 4648 4654 10.1182/blood-2006-11-056291 17289808

[B22] Santagostino E Mancuso ME Rocino A Mancuso G Mazzucconi MG Tagliaferri A Messina M Mannucci PM Environmental risk factors for inhibitor development in children with haemophilia A: a case–control study Br J Haematol 2005 130 3 422 427 10.1111/j.1365-2141.2005.05605.x 16042693

[B23] Ghirardini A Puopolo M Chiarotti F Mariani G The international registry of immune tolerance: 1994 update Vox Sang 1996 70 Suppl 1 42 6 8869468

[B24] Dimichele D The North American Immune Tolerance Registry: contributions to the thirty-year experience with immune tolerance therapy Haemophilia 2009 15 1 320 8 10.1111/j.1365-2516.2008.01880.x 18976249

[B25] Mariani G Kroner B for the Immune Tolerance Study Group (ITSG) Immune tolerance in hemophilia with factor VIII inhibitors: predictors of success Haematologica 2001 86 11 1186 1193 11694405

[B26] Schwaab R Brackmann HH Meyer C Seehafer J Kirchgesser M Haack A Olek K Tuddenham EGD Oldenburg J Haemophilia A: mutation type determines risk of inhibitor formation Thromb & Haemost 1995 74 6 1402 1406 8772209

[B27] Oldenburg J Brackmann HH Schwaab R Risk factors for inhibitor development in hemophilia A Haematologica 2000 85 10 7 14 11187876

[B28] Gouw SC van den Berg HM Oldenburg J Astermark J De Groot PG Margaglione M Thompson AR Van Heerde W Boekhorst J Miller CH Le Cessie S van der Bom JG F8 gene mutation type and inhibitor development in patients with severe hemophilia A: systematic review and meta-analysis Blood 2012 119 12 2922 34 10.1182/blood-2011-09-379453 22282501

[B29] Salviato R Belvini D Radossi P Sartori R Pierobon F Zanotto D Zanon E Castaman G Gandini G Tagariello G F8 gene mutation profile and ITT response in a cohort of Italian haemophilia A patients with inhibitors Haemophilia 2007 13 6 361 72 17610549 10.1111/j.1365-2516.2007.01437.x

[B30] Coppola A Margaglione M Santagostino E Rocino A Grandone E Mannucci PM Di Minno G AICE PROFIT,Study Group Factor VIII gene (F8) mutations as predictors of outcome in immune tolerance induction of hemophilia A patients with high-responding inhibitors J Thromb Haemost 2009 7 11 1809 1815 10.1111/j.1538-7836.2009.03615.x 19740093

